# Morbid liver manifestations are intrinsically bound to metabolic syndrome and nutrient intake based on a machine-learning cluster analysis

**DOI:** 10.3389/fendo.2022.936956

**Published:** 2022-09-06

**Authors:** Víctor Micó, Rodrigo San-Cristobal, Roberto Martín, Miguel Ángel Martínez-González, Jordi Salas-Salvadó, Dolores Corella, Montserrat Fitó, Ángel M. Alonso-Gómez, Julia Wärnberg, Jesús Vioque, Dora Romaguera, José López-Miranda, Ramon Estruch, Francisco J. Tinahones, José Lapetra, J. Luís Serra-Majem, Aurora Bueno-Cavanillas, Josep A. Tur, Vicente Martín Sánchez, Xavier Pintó, Miguel Delgado-Rodríguez, Pilar Matía-Martín, Josep Vidal, Clotilde Vázquez, Ana García-Arellano, Salvador Pertusa-Martinez, Alice Chaplin, Antonio Garcia-Rios, Carlos Muñoz Bravo, Helmut Schröder, Nancy Babio, Jose V. Sorli, Jose I. Gonzalez, Diego Martinez-Urbistondo, Estefania Toledo, Vanessa Bullón, Miguel Ruiz-Canela, María Puy- Portillo, Manuel Macías-González, Nuria Perez-Diaz-del-Campo, Jesús García-Gavilán, Lidia Daimiel, J. Alfredo Martínez

**Affiliations:** ^1^ Cardiometabolic Nutrition Group, Madrid Institute for Advanced Studies (IMDEA) Food, Excellence International Campus Autónoma Madrid University (CEI UAM) + CSIC, Madrid, Spain; ^2^ Biostatistics and Bioinformatics Unit, Madrid Institute for Advanced Studies (IMDEA) Food, Excellence International Campus Autónoma Madrid University (CEI UAM) + CSIC, Madrid, Spain; ^3^ Biomedical Research Centre for Obesity Physiopathology and Nutrition Network (CIBEROBN), Instituto de Salud Carlos III (ISCIII), Madrid, Spain; ^4^ Department of Preventive Medicine and Public Health, IdiSNA-Navarra Institute for Health Research, University of Navarra, Pamplona, Spain; ^5^ Department of Nutrition, Harvard T. H. Chan School of Public Health, Boston, MA, United States; ^6^ Biochemistry and Biotechnology Department, Nutrition Unit, Institut d’Investigació Pere Virgili (IISPV), Hospital Universitari San Joan de Reus, Universitat Rovira i Virgili, Reus, Spain; ^7^ Department of Preventive Medicine, University of Valencia, Valencia, Spain; ^8^ Cardiovascular Risk and Nutrition Research Group (CARIN), Hospital del Mar Medical Research Institute (IMIM), Barcelona, Spain; ^9^ Bioaraba Health Research Institute, Osakidetza Basque Health Service, Araba University Hospital, University of the Basque Country (UPV/EHU) , Vitoria-Gasteiz, Spain; ^10^ Department of Nursing, School of Health Sciences, Instituto de Investigación Biomédica de Málaga (IBIMA), University of Málaga, Málaga, Spain; ^11^ CIBER de Epidemiología y Salud Pública (CIBERESP), Instituto de Salud Carlos III, Madrid, Spain; ^12^ Instituto de Investigación Sanitaria y Biomédica de Alicante, Universidad Miguel Hernández (ISABIAL-UMH), Alicante, Spain; ^13^ Research Group on Nutritional Epidemiology & Cardiovascular Physiopathology (NUTRECOR). Health Research Institute of the Balearic Islands (IdISBa), University Hospital Son Espases (HUSE), Palma de Mallorca, Spain; ^14^ Lipids and Atherosclerosis Unit, Department of Internal Medicine, Lipids and Atherosclerosis Hospital Reina Sofía, Maimonides Institute for Research in Biomedicine of Cordoba (IMIBIC), Reina Sofia University Hospital, University of Cordoba, Córdoba, Spain; ^15^ Department of Internal Medicine, August Pi i Sunyer Biomedical Research Institute (IDIBAPS), Hospital Clinic, University of Barcelona, Barcelona, Spain; ^16^ Department of Endocrinology, Instituto de Investigación Biomédica de Málaga (IBIMA), Virgen de la Victoria Hospital, University of Málaga, Málaga, Spain; ^17^ Department of Family Medicine, Research Unit, Distrito Sanitario Atención Primaria Sevilla, Sevilla, Spain; ^18^ Preventive Medicine Service, Centro Hospitalario Universitario Insular Materno Infantil (CHUIMI), Canarian Health Service, Research Institute of Biomedical and Health Sciences (IUIBS), University of Las Palmas de Gran Canaria, Las Palmas, Spain; ^19^ Department of Preventive Medicine and Public Health, University of Granada, Granada, Spain; ^20^ Research Group on Community Nutrition & Oxidative Stress, University of Balearic Islands, Palma de Mallorca, Spain; ^21^ Institute of Biomedicine (IBIOMED), University of León, León, Spain; ^22^ Lipids and Vascular Risk Unit, Internal Medicine, Hospitalet de Llobregat, Hospital Universitario de Bellvitge, Barcelona, Spain; ^23^ Division of Preventive Medicine, Faculty of Medicine, University of Jaén, Jaén, Spain; ^24^ Department of Endocrinology and Nutrition, Instituto de Investigación Sanitaria Hospital Clínico San Carlos (IdISSC), Madrid, Spain; ^25^ Biomedical Research Centre for Diabetes and Metabolic Diseases Network CIBER Diabetes and Associated Metabolic Diseases (CIBERDEM), Instituto de Salud Carlos III (ISCIII), Madrid, Spain; ^26^ Department of Endocrinology, August Pi i Sunyer Biomedical Research Institute (IDIBAPS), Hospital Clinic, University of Barcelona, Barcelona, Spain; ^27^ Department of Endocrinology and Nutrition, Hospital Fundación Jimenez Díaz, Instituto de Investigaciones Biomédicas Jiménez Díaz Foundation Health Research Institute (IISFJD), University Autónoma, Madrid, Spain; ^28^ Department of Emergency, Complejo Hospitalario de Navarra Servicio Navarro de Salud-Osasunbidea, Pamplona, Spain; ^29^ Primary Care Center Cabo Huertas, Alicante, Spain; ^30^ Department of Preventive Medicine and Public Health, School of Medicine, University of Málaga, Málaga, Spain; ^31^ Department of Nutrition, Food Science and Physiology, Center for Nutrition Research, University of Navarra, Pamplona, Spain; ^32^ Nutrition and Obesity Group, Department of Pharmacy and Food Science, Lucio Lascaray Research Institute, University of the Basque Country (UPV/EHU), Vitoria, Spain; ^33^ Bioaraba Health Research Institute, Alava, Spain; ^34^ Department of Medical Sciences, University of Turin, Turin, Italy; ^35^ Nutritional Control of the Epigenome Group, Precision Nutrition and Obesity Program, Madrid Institute for Advanced Studies (IMDEA) Food, Excellence International Campus Autónoma Madrid University (CEI UAM) + CSIC, Madrid, Spain; ^36^ Department of Nutrition, Food Sciences and Physiology, University of Navarra, Pamplona, Spain

**Keywords:** hepatic enzymes, metabolic syndrome, cluster, biomarkers, glucose disorders, dyslipidemia

## Abstract

Metabolic syndrome (MetS) is one of the most important medical problems around the world. Identification of patient´s singular characteristic could help to reduce the clinical impact and facilitate individualized management. This study aimed to categorize MetS patients using phenotypical and clinical variables habitually collected during health check-ups of individuals considered to have high cardiovascular risk. The selected markers to categorize MetS participants included anthropometric variables as well as clinical data, biochemical parameters and prescribed pharmacological treatment. An exploratory factor analysis was carried out with a subsequent hierarchical cluster analysis using the z-scores from factor analysis. The first step identified three different factors. The first was determined by hypercholesterolemia and associated treatments, the second factor exhibited glycemic disorders and accompanying treatments and the third factor was characterized by hepatic enzymes. Subsequently four clusters of patients were identified, where cluster 1 was characterized by glucose disorders and treatments, cluster 2 presented mild MetS, cluster 3 presented exacerbated levels of hepatic enzymes and cluster 4 highlighted cholesterol and its associated treatments Interestingly, the liver status related cluster was characterized by higher protein consumption and cluster 4 with low polyunsaturated fatty acid intake. This research emphasized the potential clinical relevance of hepatic impairments in addition to MetS traditional characterization for precision and personalized management of MetS patients.

## Introduction

A high Metabolic Syndrome (MetS) prevalence constitutes an important clinical and public health concern in most countries (1). Moreover, the number of MetS patients is envisaged to increase in the next years (2). Indeed, the estimations about the future rates of MetS is that this condition will rise in all age groups, being associated with diverse comorbidities that could often drive an early fatal outcome (1). MetS is characterized by the presence of abdominal obesity, insulin resistance, hypertension, and hyperlipidemia as has been recognized by different organizations and institutions (3). However, other health complications have been linked to MetS development. Thus, Non-alcoholic Fatty Liver Disease (NAFLD) is associated with type 2 diabetes, obesity, metabolic and cardiovascular disorders (4, 5). The early identification of MetS accompanying factors, as well as a prescription of personalized treatment, could help to monitor these diseases with precision and will contribute to better MetS management as well as to reduce the disease impact (6). Indeed, liver steatosis is the most common phenotype of chronic hepatic disease, being linked to insulin resistance and inflammation, whose clinical stratification is still pending for personalized prescription (7).

MetS is defined by high levels of fasting blood glucose (≥100 mg/dL), high levels of triglycerides (≥150 mg/dL), high levels of blood pressure (systolic ≥130 and/or diastolic ≥85 mm Hg), central obesity and reduced high-density lipoprotein (HDL)-cholesterol concentrations (<40 mg/dL in males; <50 mg/dL) in females) (6, 8). Furthermore, higher liver enzyme levels have been associated with MetS and could be used as early indicators of this disease (9). In addition, clinical findings of frailty in MetS are associated with poorer quality of life (QoL), which have been linked to the administration of different pharmacological treatments (10), as well as polymedication, and have been associated to a worse prognosis in myocardial infarctions, hospitalization, and death in the elderly population (11). Noteworthy, the physiopathology of NAFLD may involve diverse endocrine disorders that need to be understood as well as the specifical clinical or hormonal complications related to MetS and the role of nutrition in NAFLD prevention and management (12).

Nowadays, patients with MetS are currently prescribed with several pharmacological agents as well as advice with significant nutritional and lifestyle style recommendations (13). The most usual advice with such purpose has been mainly to increase physical activity and follow a healthy dietary pattern such as the Mediterranean Diet (MedDiet) or other similar balanced diets (14). However, MetS components are generally treated individually, and different grades of this disease are merely classified according to the number of fulfilled MetS criteria without considering other variables that could be involved in the onset and development of this condition. Thus, regular clinical management does not perform the required personalization or interindividual differential responses to each intervention (15), which should be encouraged in term of individualized medicine. In this context, proteins, fatty acids and antioxidants are recognized nutrients involved in MetS development and liver morbidity (16). Likewise, the management of MetS features may include the prescription of specific dietary recommendations such as low carbohydrate diet or low-fat diets, being fundamental a tailored advice for each patient situation (17) to exacerbate adverse clinical manifestation (18).

In this context, precision medicine has been described as a key strategy to provide better health advice and wellbeing (19). The identification and monitoring of different clusters of patient, with specific clinical features, could help in the prognosis and targeted management with individualized advice (20). Personalized medicine could not only improve the efficiency of medical advice, but may also be able to overcome the barriers that this type of medicine has in general practice (21). The integration of different “omics” and bioinformatic/statistical tools to analyze patients’ features will provide a wider picture of the specific health needs of each individual (22). Moreover, current endeavors of multiple scientific societies include the reclassification of groups of patients with common characteristics for a more precise, effective and faster treatment (23–25).

The aim of this study was the design of a classification of MetS patients with high cardiovascular risk considering clinical issues and pharmacological therapies, as well as biochemistry and anthropometric markers that can be easily collected in regular anamnesis in clinical practice with a diagnosis, prognosis and impact on personalized management. In addition, a comparison concerning nutrient intake depending on cluster between each group of patients.

## Material and methods

### Study population

The population sample studied in this cross-sectional research belongs to the PREDIMED-Plus trial (26), specifically subjects at high metabolic risk. This project is a controlled, randomized, multicenter (23 recruitment centers) clinical trial of 6 years of intervention plus 2 years of follow-up. The primary aim is to assess the effect of intensive intervention for weight loss based on an energy-restricted Mediterranean diet (er-MedDiet), physical activity and behavioral support (intervention group) to prevent major cardiovascular events in comparison with a control group following conventional primary care for prevention of cardiovascular disease. The full protocol, including recruitment strategy, randomization procedure and intervention has been previously reported and is available at http://predimedplus.com/ (26). This investigation has been approved by the Institutional Review Boards (IRBs) of all participating centers according to the ethical standards of the Declaration of Helsinki.

**Figure 1:**
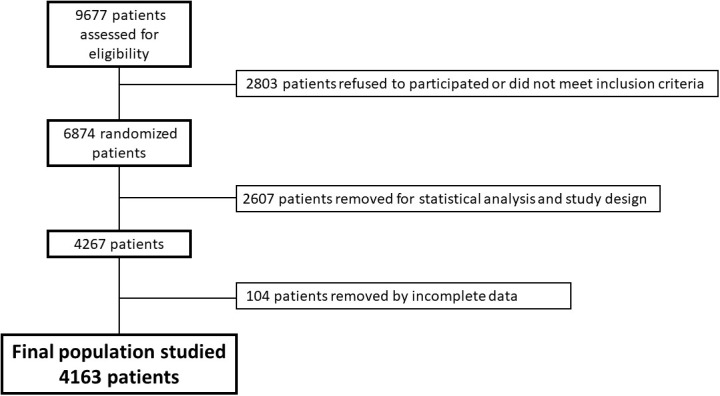
Flowchart of selection of studied sample.

Participants are men (55-75 years) and women (60-75 years) with overweight/obesity (BMI 27-<40 Kg/m^2^) who met at least three MetS criteria (8). This trial was retrospectively registered at the International Standard Randomized Controlled Trial Registry with the number 89898870. The study sample included only basal data before administering any intervention to participants. Volunteers with missing values in the selected variables (**Supplementary Table 1**) were excluded, as well as patients with a deviation of the variable’s values 3 times more than the interquartile range. The final number of participants included in the analysis was 4163 participants (**Figure 1**).

### Variable selection and categorization approach

A MetS Score has been estimated with the sum of the relative Z-scores of body mass index (BMI), HDL-cholesterol, triglycerides, systolic pressure and glucose levels according to the National Cholesterol Education Program’s Adult Treatment Panel III (ATP III)  (27). The adherence to an er-MedDiet was assessed with a validated specific screener designed for the PREDIMED-Plus intervention (28). The er-MedDiet score was calculated using the sum of positive answers obtained from the 17-item MEDAS questionnaire (28). The Neutrophil-lymphocytes Index (NLI) was calculated using the quotient between neutrophils and lymphocytes levels obtained in the hemogram (29).

The dietary intake assessment of participants was carried out using a validated semi-quantitative 143-item FFQ (30–32) able to reflect differences in dietary patterns and seasonal variability. Participants answered the average consumption of a commonly used portion size (e.g., glass, cup, slice) for each food or beverage item. Daily consumption for each food was calculated using the portion size multiplied by the frequency of consumption and then expressed as grams per day. This estimation was not possible for the fried foods item as the portion size is not specified in the FFQ. Mini-Mental State Examination (MMSE) test and Beck depression questionnaire were used as an outcome of differences between the group of patients (33–35).

A panel of variables was categorized to clustered MetS patients. The selected markers included anthropometric variables (waist-hip ratio and blood pressure), biochemistry variables (glucose, HDL-cholesterol, LDL-cholesterol, cholesterol, NLI and platelets), hepatic enzymes (Aspartate aminotransferase (AST), Alanine Aminotransferase (ALT), Gamma-glutamyl transferase (GGT)) and pharmacological treatments. The criteria followed for the selection of variables was the combination of well-established MetS criteria (anthropometric variables, glucose, total cholesterol, HDL-cholesterol and LDL-cholesterol) (8) with other markers that are commonly altered in the comorbidities associated with MetS. In this regard, platelets were selected due to their relationship with low degree inflammation (36). NLI has been described before as biomarker of inflammation in cardiovascular disease and this biomarker could have relevant importance in low grade inflammation (29, 37) while liver enzymes have been postulated as valuable indicators of MetS and cardiovascular disease (38). Finally, pharmacological treatments related to the well-established polymedication with worse MetS prognosis were accounted for (11). In this context, the distribution of variables was carried out following well-defined risk criteria (**Supplementary Table 1**). In addition, categorization of hypertension, glucose, LDL-cholesterol and total cholesterol was carried out using a combination of basal measurements of these parameters with specific medication for each morbidity (antihypertensives, metformin, statins and cholesterol medication) to discern non-controlled patients taking medication.

### Statistical Procedures

Description of the population was made according to the MetS score calculated for each participant. MetS Score has been calculated with the sum of Z-scores of BMI, HDL-cholesterol, triglycerides, systolic pressure and glucose levels (MetS Score = ((HDL - mean HDL)/SD HDL) + ((BMI- mean BMI)/SD BMI) + ((TG- mean TG)/SD TG) + ((Diastolic BP-mean Diastolic BP)/SD Diastolic BP + ((Glucose-mean Glucose)/SD Glucose) (39). The population has been classified in Low MetS Score (score lower than the median) and High MetS Score (score higher than the median).

Participants were classified as low (score ≤ 4.97 MetS score) or high (> 4.97 MetS score). Statistical differences between groups were assessed by t-Student test, to determine the differences between patients with lower and higher MetS score, or analysis of variance (ANOVA) for quantitative variables, and chi-squared was implemented for categorical variables.

### Exploratory factor analysis and clusterization

An stepwise exploratory factor analysis was carried out on the remaining variables to remove those with low adequacy, which was assessed with the Kaiser-Meyer-Olkin score and variables with an unacceptable score below 0.5 were discarded (40). Studied variables were selected by a stepwise exploratory factor analysis which allows the identification of latent variables and accounts for the distribution of each variable contributes to each factor among a large number of subjects. A final set of 17 variables was finally selected to perform the subsequent cluster analysis (**Table 2**). A factor analysis was performed to determine the number of factors that provide the higher Tucker Lewis Index of factoring reliability (> 0.8) and a RMSEA index (<0.05) that indicates a good fit to the data. Variables included in the final panel were those presenting a loading factor higher than 0.25, in absolute values. These variables were considered representative contributors to each latent variable estimated from the analysis. For each patient, a factor score was computed from the obtained factor of the final solution. Such scores were used to perform a hierarchical cluster analysis to discern different patterns of patients according to the weight that every latent variable has for each patient. Clustering analysis was performed using Ward’s hierarchical clustering method (41, 42). The cut point for the cluster identification was achieved using the “factoextra” R package function to determine and visualize the optimal number of clusters using different methods: within-cluster sums of squares, average silhouette and gap statistics (43) being gap statistics the optimal approximation. ANOVA between clusters was adjusted by age, sex and recruitment center, whereas qualitative variables were analyzed using Chi-Square test.

All the statistical analyses were performed using the R programming language (44) with the RStudio tool (45). The following R libraries were used to carry out all the statistical approaches: “Hmisc”, “psych”, “factoextra”, “ggplot2”, and “**Table 1**”.

**Table 1:** Baseline characteristics of patients regarding MetS components.

	Low MetS score	High MetS score	*p*-value
	(N = 2082)	(N = 2081)	
**Age (years)**	65.0 (4.96)	65.5 (4.81)	0,002
**BMI^1^ (Kg/m2)**	31.0 (2.76)	34.0 (3.38)	<0.001
**Waist-Hip ratio**	0.981 (0.0728)	0.981 (0.0789)	0,872
**Systolic blood pressure (mmHg)**	133 (14.2)	146 (16.9)	<0.001
**Diastolic blood pressure (mmHg)**	78.6 (9.26)	82.7 (10.1)	<0.001
**Glucose (mg/dl)**	102 (14.8)	119 (24.3)	<0.001
**Uric Acid (mg/dl)**	5.97 (1.43)	5.98 (1.45)	0,832
**Cholesterol (mg/dl)**	188 (33.7)	198 (36.6)	<0.001
**HDL cholesterol (mg/dl)**	45.7 (9.95)	50.0 (12.0)	<0.001
**LDL cholesterol (mg/dl)**	118 (30.3)	117 (32.3)	0,87
**NLI^2^ (p.d.u.)**	1.91 (0.816)	1.92 (1.59)	0,823
**Platelets (10^3^/µL)**	229 (55.3)	235 (56.0)	<0.001
**AST (U/L)**	21.8 (5.93)	22.0 (6.44)	0,48
**ALT (U/L)**	23.9 (9.66)	25.7 (10.8)	<0.001
**GGT (U/L)**	28.1 (14.4)	32.1 (16.2)	<0.001
**Beck Depression questionnaire Score**	7.93 (7.01)	9.00 (7.54)	<0.001
**Mini-Mental questionnaire Score**	28.3 (1.79)	28.1 (2.06)	<0.001
**erMedDiet^3^ 17 points**	8.47 (2.69)	8.46 (2.61)	0,879
**Sedentary lifestyle (Yes %)**	1211 (58.2%)	1130 (54.3%)	0,012
**MetSscore**	-1.71 (1.15)	1.71 (1.44)	<0.001

^1^BMI, Body Mass Index ^2^ Neutrophils- Lymphocytes Index ^3^erMedDiet, Mediterranean Diet Adherence questionnaire 17 points Score.

## Results

### Baseline characteristics

The mean age of the sample was 65.2 years old (**Table 1**). Significant differences in all MetS components between high MetS and low MetS were found. Participants in the High MetS Score group showed significantly higher scores in the Beck Depression Questionnaire Score and lower scores in the Mini-Mental State Exam Questionaries (MMSE). However, no differences in adherence to an erMedDiet were found between groups, although participants with High MetS Score were less sedentary. There were no relevant differences in levels of LDL-cholesterol, uric acid, NLI and AST.

### Factor scores: Association between selected variables

A factor analysis was carried out using a stepwise selection of variables that were relevant in MetS and variables that were easily collected during regular health check-ups (**Supplementary Table 1**). The factor analysis enabled to define three main factors (**Table 2**) with a Tucker Lewis Index of factoring reliability of 0.887 and a RMSEA index of 0.034. The first factor was characterized by the role of cholesterol and LDL-cholesterol treatments and serum levels; the second factor was associated with glucose metabolism impairments and accompanying treatments, and the third factor was driven by changes in hepatic enzymes.

**Table 2:** Factor loading of pattern matrix.

	Factor 1	Factor 2	Factor 3
**Variance proportion**	0.42	0.32	0.26
**Aspirin use**	0.00	0.24	-0.03
**Pain treatment**	0.06	-0.02	-0.02
**Tranquilizers use**	0.06	-0.01	-0.05
**CVD treatment**	-0.04	0.04	-0.03
**No metformin diabetes treatment**	0.00	**0.84**	-0.02
**Insulin use**	0.00	**0.36**	-0.06
**HDL cholesterol**	0.19	-0.07	0.02
**Waist-Hip ratio**	-0.05	0.05	0.04
**Hypertension**	0.02	-0.08	0.09
**Cholesterol**	**0.90**	0.02	0.00
**Glucose**	-0.01	**0.76**	0.03
**LDL cholesterol**	**0.52**	-0.12	-0.02
**NLI Index**	-0.11	0.10	-0.03
**Platelets**	0.08	0.05	-0.02
**AST**	-0.01	0.00	**0.51**
**ALT**	0.00	0.00	**0.77**
**GGT**	0.05	0.01	**0.31**

Bold values means most relevants factor loadings values (upper than 0.25).

### Cluster analysis

**Figure 2:**
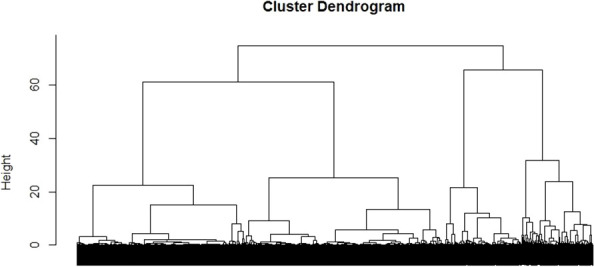
Euclidean tree for patient's clusterization.

Using the z-scores obtained in the Factor Analysis, a hierarchical cluster analysis was carried out to classify different groups of patients according to the loading factors of these variables. The cluster analysis allowed for the definition of four different clusters (**Supplementary Figure 1** and **Figure 2**).

The analysis of factor loadings between each cluster (**Table 2**) revealed that cluster 1 is characterized by higher levels of factor 1 associated variables(cholesterol and LDL-cholesterol treatments and these biochemistry markers levels), cluster 2 did not present remarkable differences in any factor, cluster 3 presented higher levels concerning factor 3 associated variables (hepatic enzymes) and cluster 4 highlighted factor 3 variables (glucose metabolism disorders associated parameters).

**Figure 3:**
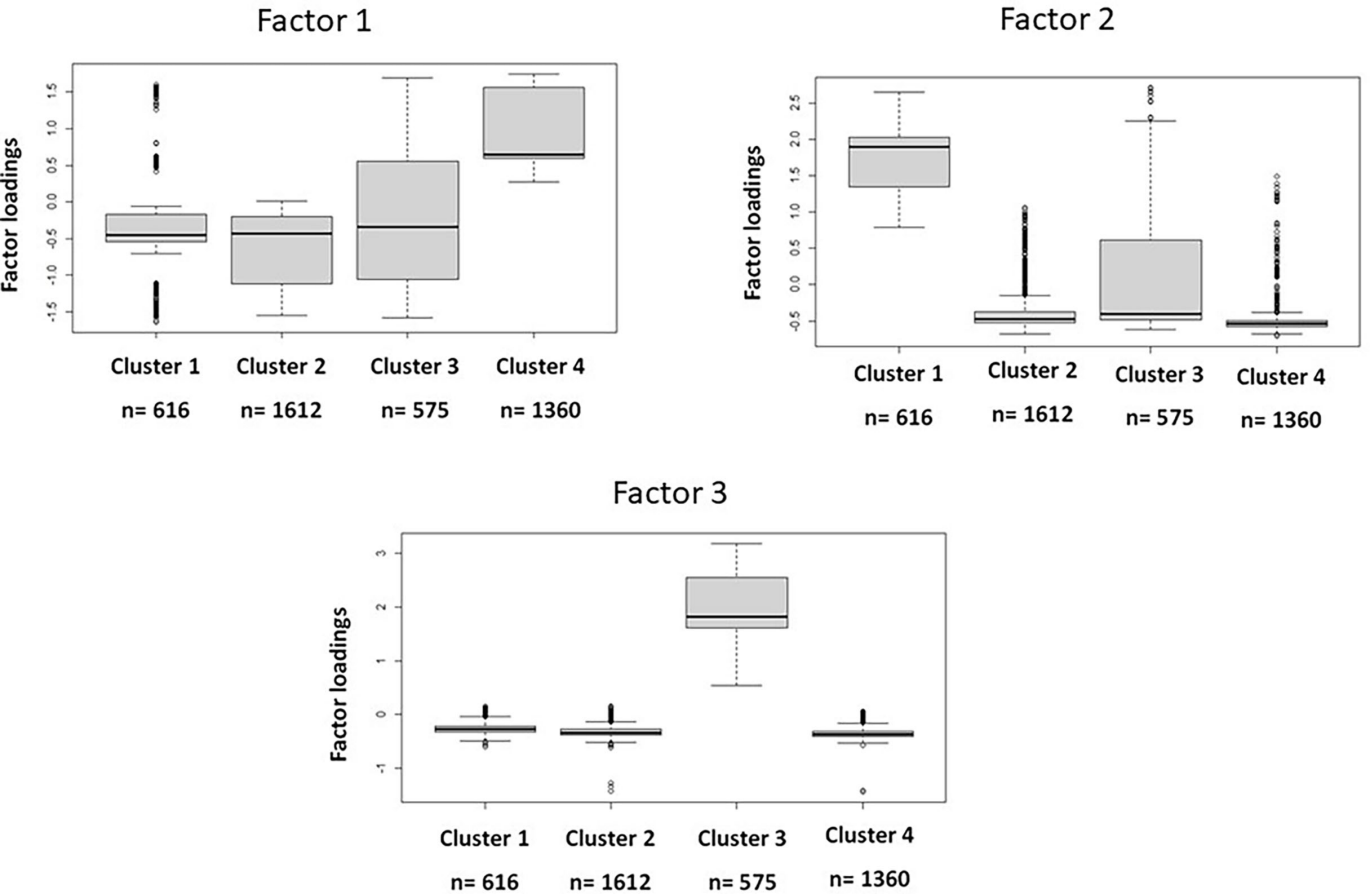
Differences between cluster for each factor.

Comparisons between the weight of the factors in each cluster (**Figure 3**) confirmed that factor 1 variables were highlighted in patients belonging to cluster 4 factor 2 variables are more relevant in cluster 1, variables of factor 3 have more importance in cluster 3, and patients of factor 2 all variables were prominent.

The analysis of patients’ characteristics (**Table 3**) revealed an unequal patient distribution, especially in clusters 1 and 3, where the number of patients that belong to these clusters (616 and 575 patients respectively) is half of the other two clusters. Regarding anthropometric and psychosocial measures between each cluster, differences in sex distribution showed that cluster 3 has a higher percentage of men while cluster 4 has a higher percentage of women.

**Table 3:** Differences between clusters in anthropometric, psychosocial, biochemistry and disease prevalence parameters adjusted by sex, age and recruitment center.

	Cluster 1	Cluster 2	Cluster 3	Cluster 4	*p*-value
	(N = 616)	(N = 1612)	(N = 575)	(N = 1360)	
**Sex (%)**					
Men	51.5	55.8	65.7	36.7	<0.001
Women	48.5	44.2	34.3	63.3	
**Age (years)**	65.8 (4.61)	65.9 (4.96)	63.1 (4.73)	65.2 (4.75)	<0.001
**BMI^1^ (Kg/m^2^)**	32.6 (3.52)	32.4 (3.44)	32.8 (3.36)	32.3 (3.40)	0.493
**Waist-Hip Ratio**	1.00 (0.07)	0.98 (0.07)	1.01 (0.07)	0.96 (0.07)	<0.001
**Diastolic blood pressure (mmHg)**	139 (17.5)	139 (17.7)	140 (16.2)	139 (16.3)	0.987
**Systolic blood pressure (mmHg)**	78.2 (9.86)	80.2 (9.97)	82.2 (9.56)	81.6 (9.72)	<0.001
**MetS score**	5.18 (0.514)	4.83 (0.419)	5.07 (0.476)	5.09 (0.430)	<0.001
**Glucose (mg/dl)**	134 (26.3)	106 (17.0)	116 (23.8)	104 (15.3)	<0.001
**Hba1c**	6.83 (0.901)	5.88 (0.535)	6.17 (0.777)	5.84 (0.494)	<0.001
**Cholesterol (mg/dl)**	173 (32.1)	172 (19.7)	189 (36.4)	228 (22.2)	<0.001
**HDL (mg/dl)**	45.6 (10.8)	45.8 (10.3)	45.5 (10.4)	52.2 (11.6)	<0.001
**LDL (mg/dl)**	99.5 (28.2)	101 (19.0)	114 (32.5)	146 (21.8)	<0.001
**Triglycerides (mg/dl)**	141 (55.3)	128 (48.9)	148 (54.2)	146 (52.0)	<0.001
**AST (U/L)**	19.2 (4.44)	20.7 (4.58)	31.2 (6.99)	20.6 (4.39)	<0.001
**ALT (U/L)**	21.6 (6.63)	21.8 (6.50)	44.0 (8.70)	21.6 (6.28)	<0.001
**GGT (U/L)**	28.7 (14.2)	27.7 (14.1)	41.2 (18.1)	28.9 (14.4)	<0.001
**Platelets (10^3^/µL)**	242 (61.7)	225 (54.8)	228 (52.7)	237 (54.1)	<0.001
**NLI^2^ (p.d.u.)**	2.16 (2.69)	1.95 (0.85)	1.83 (0.67)	1.79 (0.74)	<0.001
**Uric acid (mg/dl)**	5.79 (1.37)	5.99 (1.42)	6.21 (1.49)	5.94 (1.45)	0.128
**erMedDiet ^3^ 17 Points**	8.78 (2.51)	8.37 (2.60)	8.16 (2.71)	8.56 (2.72)	0.472
**Sedentary lifestyle (Yes %)**	45.1	43.5	52.3	39.8	<0.001
**Beck Depression questionnaire Score**	9.40 (8.21)	8.00 (6.83)	8.11 (7.33)	8.74 (7.34)	0.948
**Mini-Mental questionnaire Score**	27.9 (2.20)	28.3 (1.80)	28.4 (1.78)	28.2 (2.00)	0.008
**Diabetes prevalence (Yes %)**	603 (97.9%)	227 (14.1%)	170 (29.6%)	96 (7.1%)	<0.001
**Hypertension Prevalence (Yes %)**	538 (87.3%)	1386 (86.0%)	493 (85.7%)	1123 (82.6%)	<0.001
**Dyslipidemia prevalence (Yes %)**	481 (78.1%)	1095 (67.9%)	389 (67.7%)	935 (68.8%)	<0.001

p-value refers to chi-squared and ANOVA test comparisons between clusters. ^1^BMI, Body Mass Index ^2^Neutrophils- Lymphocytes Index ^3^erMedDiet, Mediterranean Diet Adherence questionnaire 17 points Score.

Regarding anthropometric variables, the waist-hip ratio was significantly different between clusters whereas BMI did not present any statistical difference. Moreover, systolic blood pressure was also different between clusters whereas diastolic blood pressure was not. Biochemistry measurements showed important significant differences between clusters, except for uric acid. Beck questionnaire and Mini-Mental questionnaire scores did not evidence significant discrepancies; however, sedentarism levels were different in cluster 3. Finally, regarding to reported disease prevalence, patients in cluster 1 have a higher diabetes and dyslipidemia prevalence while reported hypertension is similar in the 4 clusters.

**Figure 4:**
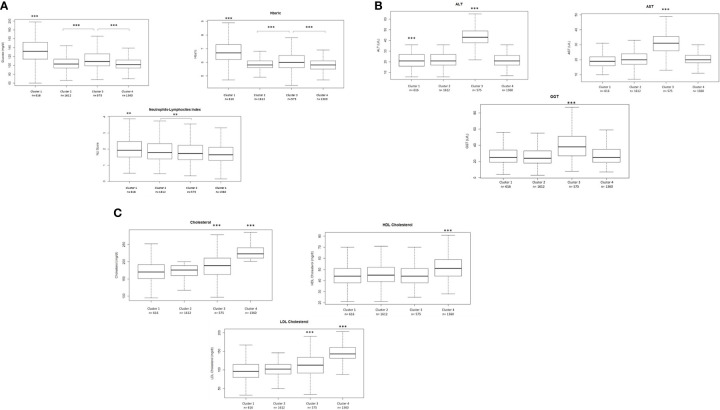
**(A)** Differences in glucose. Hba1c and NLI between clusters. **(B)** Differences in transaminases levels between clusters. **(C)** Differences in total cholesterol. HDL-cholesterol and LDL- cholesterol levels between clusters. Comparisons were carried out using a Student's t-test adjusted by the Bonferroni posthoc test. ***p<0.001 **p<0.01.

The analysis of the variables differentially representative in each cluster showed that in cluster 1, levels of glucose and glycosylated hemoglobin are higher compared to the rest of the clusters (**Figure 4A**). Additionally, cluster 3 presents also higher levels compared to cluster 2 and cluster 4. Moreover, values of NLI are elevated in cluster 1 compared to the other clusters. On the other hand, patients belonging to cluster 3 were characterized by alterations of transaminase levels, which are higher in comparison to the other clusters, while cluster 1 presented lower levels (**Figure 4B**). Cluster 4 highlighted that cholesterol levels (total cholesterol, HDL and LDL) were significantly higher than those found in the patients of the other clusters (**Figure 4C**).

Additionally, we performed the last comparison between clusters regarding energy and nutrients intakes (**Table 4**). In this sense, significant differences in intake of protein, polyunsaturated fatty acids, linoleic acid, vitamin A and vitamin E were found among clusters.

**Table 4:** Differences between clusters in energy and nutrient’s intake adjusted by sex, age and recruitment center.

	Cluster 1	Cluster 2	Cluster 3	Cluster 4	p value
	(N = 616)	(N = 1612)	(N = 575)	(N = 1360)	
**Total energy intake (kcal)**	2300 (523)	2390 (559)	2450 (577)	2310 (544)	0.34
**Carbohydrates (g/day)**	233 (69.5)	247 (73.3)	251 (72.4)	240 (72.8)	0.63
**Protein (g/day)**	97.3 (22.7)	97.5 (22.2)	99.4 (22.3)	95.5 (21.8)	0.03
**Total fat (g/day)**	101 (27.9)	104 (29.1)	106 (29.9)	101 (28.0)	0.20
**Monounsaturated fat (g/day)**	52.0 (16.1)	53.8 (16.7)	54.6 (16.7)	52.2 (15.6)	0.37
**Polyunsaturated fat (g/day)**	17.8 (6.76)	18.2 (6.77)	18.4 (7.11)	17.4 (6.47)	0.02
**Saturated fat (g/day)**	25.4 (8.02)	26.4 (8.42)	27.1 (8.81)	25.3 (8.22)	0.13
**Trans fat (g/day)**	0.57 (0.37)	0.61 (0.40)	0.63 (0.43)	0.56 (0.36)	0.14
**Linoleic acid (g/day)**	13.6 (5.78)	13.8 (5.69)	13.8 (5.75)	13.2 (5.57)	0.02
**Linolenic acid (g/day)**	1.39 (0.652)	1.47 (0.697)	1.44 (0.659)	1.40 (0.666)	0.24
**Ω-3 Fatty acid (g/day)**	0.88 (0.48)	0.89 (0.47)	0.88 (0.44)	0.88 (0.49)	0.96
**Alcohol (g/day)**	10.1 (15.1)	10.6 (14.4)	13.3 (16.4)	9.27 (13.6)	0.12
**Fiber (g/day)**	26.6 (8.96)	26.5 (8.90)	25.8 (8.79)	26.3 (8.74)	0.25
**Vitamin A (mcg/day)**	1150 (697)	1110 (622)	1080 (643)	1070 (622)	0.03
**Vitamin C (mg/day)**	202 (84.6)	207 (87.4)	193 (84.7)	202 (84.3)	0.28
**Vitamin E (mg/day)**	10.9 (4.49)	10.7 (3.94)	10.5 (4.00)	10.4 (3.87)	0.01

p-value refers to ANOVA test comparisons between clusters.

## Discussion

In this study, the clinical status of patients over 55 years old diagnosed with MetS was assessed and categorized to explore potential clusterized subgroups with potential precision management. We obtained that these patients could be categorized in 4 different clusters depending on the MetS profile and severity. For that purpose, a selection of 17 variables was performed which are easily accessible in a routine health check-up. While some of these variables are commonly used in MetS characterization such as waist circumference, blood pressure, glycemia levels or circulating HDL-cholesterol levels (8), other new variables were incorporated that have been demonstrated to be relevant in the context of this disease. Liver enzymes have been included to perform factor and cluster analysis because levels of ALT are positively correlated with MetS prevalence or with accompanying morbid components in a Korean adult population (46). Moreover, research conducted by Tsao Y. et al. (47) reported that higher levels of ALT and GGT are associated with higher risk of MetS in an indigenous population in Taiwan; furthermore, it has been described that the AST-ALT ratio can be used as a potential marker of MetS risk (48). Physical activity/condition and dietary habits were not included in the analyses due to not having enough statistical relevance in this model and they could be considered a cause or consequence of MetS alterations but, interestingly, a non-significant trend was observed to a higher score in the erMedDiet questionnaire in the population with high MetS. A reason for this inverse causality could be that people with worse health status received nutritional advice before the start of this study, considering that MedDiet is one of the first health interventions for this type of patient (49). Lastly, high levels of platelets are associated with inflammatory processes (50, 51). Considering it has been widely described that MetS produces a chronic low-grade systemic inflammation (52, 53), the monitorization of markers related to inflammation could be a great opportunity to carry out a precision management of MetS (54).

MetS is a condition that is more prevalent with the increase of age (55). Age-related diseases are also associated with poor quality of life that has, consequently, the necessity of pharmacological treatment for depression and physical pain, resulting in a frequent coexistence between MetS and depression or pain in this type of patient (56). This type of medication in MetS patients management is controversial and its use may produce other unwanted secondary effects due to the high number of interactions that these drugs elicit (57). Moreover, categorizing patients depending on their risk levels and the use of drugs allows for a better approach about how the patient’s complications are receiving a good pharmacological management of these alterations and it could be an easy tool to be used in clinical practice, saving time for the patients diagnose. For example, patients with resistant hypertension could have more risk of developing cardiovascular events, as well as being associated with hyperaldosteronism and obstructive sleep apnea (58). Moreover, irregular management of blood glucose levels and high percentages of glycosylated hemoglobin could produce an increase in cancer, mental and nervous system disorders, infections and liver disease (59), so a carefully prescribed pharmacological approach must be mandatory, especially in patients which show ineffective treatment, which may be supported by patients clusterization.

The performed cluster analysis showed that patients diagnosed with MetS (at least 3 of 5 of MetS criteria) could be subclassified into four groups according to other clinical manifestations. First, cluster 2 presents mildly adverse biochemistry parameters closer to normality than the other clusters, which could indicate that this specific group of patients, despite being diagnosed with MetS, are healthier than the others. However, these differences are not reflected in differences in BMI, blood pressure or the er-MedDiet score. This suggests that adherence to the Mediterranean diet, due to inverse causality, may not have an important effect in this classification of MetS profile, but could have an influence on the treatment of these patients.

Furthermore, the analysis showed the presence of two clusters that elicit more morbid manifestations. On one hand, cluster 1, which suggested a glucose metabolism impairment expressed as higher levels of glucose and glycosylated hemoglobin. The higher alterations in these measurements could suggest that this cluster of patients has more cardiometabolic disorders than the patients belonging to the other clusters (60). The clinical management of these patients must be more intense due to hyperglycemia has been associated with increased risk of dementia and mental disorders in the elderly population depending on the APOE genotype (61). Moreover, cluster 1 also presents a non-significant worse tendency in the Beck Depression questionnaire score and Mini-Mental questionnaire score, which could indicate that mood and quality of life alterations could be related to hyperglycemia. Cluster 1 is also marked by a high ratio of NLI. The increment of NLI levels has been described to be an early predictor of cardiovascular events (62), acute myocardial infarction in type-2 diabetes patients (63), or a marker of systemic inflammation (64). Thus, Surendar J. et al. (65) described in an Indian population (CURES-143) a direct and positive relation between MetS number of criteria and NLI score suggesting that NLI could be a great indicator of MetS severity and higher metabolic disorders. Moreover, in a Turkish population, such relationship has been also described (66). However, more studies are needed in Caucasian populations to confirm that these findings are not dependent of race.

In this sense, cluster 2 is clinically characterized by elevated cholesterol levels, which are a well-established indicator of MetS (8). Glucose and cholesterol disorders are usually related in MetS patients, but this analysis has shown that these patients could be discriminated regarding these alterations. A precision approach could be envisaged as a key point in the future management of MetS.

Finally, the current analysis raised a cluster (cluster 3) characterized by relevant impaired hepatic indicators. In addition to impaired glucose metabolism, cluster 3 presents an altered transaminases profile that could indicate a certain level of liver injury as is reflected in the FLI score. Elevated levels of transaminases with alterations in blood glucose levels are commonly associated with nonalcoholic fatty liver disease (NAFLD) (67) that could derive to liver fibrosis. These outcomes would suggest the presence of early steps of metabolic associated fatty liver disease (MAFLD) that has been described to appear as a frequent consequence of MetS development (68, 69), indicating that its severity must be immediately controlled.

This cluster analysis shows the heterogeneity that MetS could present depending on patients’ characteristics and morbidity. Similar analysis has demonstrated their utility in other cardiometabolic diseases such as Atrial Fibrillation (AF) (70) or Sickle cell anemia (71).

The analysis of differences in nutrient intake between clusters indicates differences in protein, polyunsaturated fatty acids, linoleic acid and some vitamins (A and C). Cluster 3, that have altered hepatic parameters, presents a higher consumption of protein. Accordingly, protein intake has been reported to be inversely correlated with the risk of NAFLD incidence in men and women aged ≥50 years (72). These data suggest that high protein intake may be one of the origins of liver enzyme alterations.

On the other hand, cluster 4, which presents more altered lipid profile parameters has the lowest consumption of polyunsaturated fatty acids and linoleic acids compared to the other cluster. Dietary PUFA intake is a beneficial factor for dyslipidemia (73) and its low consumption may be related to a worse lipid profile.

Finally, the consumption of well-recognized antioxidants vitamin A and vitamin E, is lower in clusters 3 and 4 than in the other groups. Practical nutritional guidelines usually recommend the intake of antioxidants vitamins in MetS-associated diseases (74). Vitamin A is positively associated with metabolic risk factors in Chinese children and adolescents (75), while vitamin E levels are significantly lower in MetS patients (76). Thus, MetS worse prognosis may be linked to a low intake of antioxidant vitamins, but further studies are needed to confirm this association. Overall, the liver status statement is relevant in high cardiovascular risk patients as well as the role of precision nutrition for the management of metabolic syndrome features (77).

To conclude, this research demonstrated a new assessment of MetS patients based on markers that are commonly used in clinical practice but are not used in MetS management. This approach has been able to identify the potential impact of an altered hepatic function profile in MetS for a differential prognosis and therapies. These subgroups of patients represent the variability between characteristics that MetS patients could have and may be relevant in the study of complications associated with the development of MetS as well as different nutritional dietary patterns have been found between groups. Further research must be conducted in order to confirm the potential of this patient’s classification and to anticipate the possible disease evolution of such population. In any case, the involvement of medications and liver status assessments reveals that current metabolic syndrome categorization may need to be re-appraised. This approach could be a practical clinical tool to provide the most adequate lifestyle and behavioral intervention depending on the subgroup of patients and their metabolic status for better personal and precise management of this disease. Our result confirms the clinical significance of liver status in Metabolic Syndrome characterization to understand and predict the prognosis of that disease for personalized precision medicine. Indeed, a cluster analysis based on metabolic biomarkers revealed the potential importance of pivotal liver morbidity manifestations, which are associated with nutrient intake in MetS patients.

## Data availability statement

The PREDIMED-Plus trial enclosed restrictions on data availability because of the signed consent agreements around data sharing. These only enable access to external researchers for studies following the project purposes. PREDIMED_Plus trial access request of data used in this study can make a request to the PREDIMED-Plus trial Steering Committee chair: jordi.salas@urv.cat. The request will then be passed to members of the PREDIMED-Plus Steering Committee for deliberation. Requests to access the datasets should be directed to PREDIMED-Plus trial Steering Committee chair: jordi.salas@urv.cat.

## Ethics statement

This study has been reviewed and approved by the different ethics committee of each recruitment center: CEI Provincial de Málaga-Servicio Andaluz de Salud O01_feb_PR2 - Predimedplus nodo 1 CEI de los Hospitales Universitarios Virgen Macarena y Virgen del Rocıó Servicio Andaluz de Salud PI13/00673 CEIC Universidad de Navarra 053/2013 CEI de las Illes Balears - Conselleria de Salut Direcció General de Salut Publica i Consum IB 2242/14 PI CEIC del Hospital Clınic de Barcelona HCB/2016/0287 CEIC Parc dé Salut Mar y IDIAP Jordi Gol PI13/120 CEIC del Hospital Universitari Sant Joan de Reus y IDIAB Jordi Gol 13-07-25/7proj2 CEI de la Provincia de Granada- Servicio Andaluz de Salud MAB/BGP/pg CEIC pf Fundacion Jiménez Dıaz EC 26-´ 14/IIS-FJD CEIC Universidad de Navarra 053/2013 CEIC Euskadi PI2014044 CEIC Corporativo de Atención Primaria de la Comunitat Valenciana 2011-005398-22 CEI Humana de la Universidad de las Palmas de Gran Canaria CEIH-2013-07 CEIC del Hospital de Bellvitge PR240/13 CEI de Cordoba-Junta de Salud 3078 CEI of Fundación IMDEA Alimentación PI-012 CEIC Hospital Clınico San Carlos de Madrid-Piloto-CEIĆ Servicio Madrileño de salud-General 30/15 CEI Provincial de Málaga-Servicio Andaluz de Salud CEI de las Illes Balears - Conselleria de Salut Direcció General de Salut Publica i Consum IB 2251/14 PI CEIC del Hospital Clınic de Barcelona HCB/2017/ ´ 0351 CEIC del Hospital General Universitario de Alicante CEIC PI2017/02 CEIC de la Investigación Biomédica de Andalucıá (CCEIBA) CEI de la Universidad de León é TICA-ULE-014- 2015. The patients/participants provided their written informed consent to participate in this study.

## Author contributions

MAM-G, DC, JS-S, MF, AA-G, JW, JV, JVd, DR, JL-M, RE, FT, JL, JL-M, AB-C, JT, VMS, XP, MD-R, PM-M, JV, JVd, CV, JG-G, MM-G, AG-R, NB, LD and JAM designed and conducted the research. VM, RSC, and JAM conceived the study idea and the analysis design. JAM supervised the research and VM carried out the analysis procedures, bibliographic research, data preparation, statistical analysis and wrote initial drafts. VM and RS-C assisted with statistical analysis and R programming. VM, RS-C, RM, LD NPDdC and JAM participated in the scientific discussion of experimental results. All the authors assisted in manuscript revision for intellectual content and approved it.

## Funding

The PREDIMED-Plus trial was supported by the European Research Council (Advanced Research grant 2014–2019; agreement #340918; granted to MM-G); the official Spanish institutions for funding scientific biomedical research, CIBER Fisiopatología de la Obesidad y Nutrición (CIBEROBN) and Instituto de Salud Carlos III (ISCIII) through the Fondo de Investigación para la Salud (FIS) which is co-funded by the European Regional Development Fund (coordinated FIS projects led by JS-S and JV, including the following projects: PI13/00673, PI13/00492, PI13/00272, PI13/01123, PI13/00462, PI13/00233, PI13/02184, PI13/00728, PI13/01090, PI13/01056, PI14/01722, PI14/00636, PI14/00618, PI14/00696, PI14/01206, PI14/01919, PI14/00853, PI14/01374, PI14/00972, PI14/00728, PI14/01471, PI16/00473, PI16/00662, PI16/01873, PI16/01094, PI16/00501, PI16/00533, PI16/00381, PI16/00366, PI16/01522, PI16/01120, PI17/00764, PI17/01183, PI17/00855, PI17/01347, PI17/00525, PI17/01827, PI17/00532, PI17/00215, PI17/01441, PI17/00508, PI17/01732, PI17/00926, PI19/00957, PI19/00386, PI19/00309, PI19/01032, PI19/00576, PI19/00017, PI19/01226, PI19/00781, PI19/01560, PI19/01332, PI20/01802, PI20/00138, PI20/01532, PI20/00456, PI20/00339, PI20/00557, PI20/00886, PI20/01158), and the Especial Action Project “Implementación y evaluación de una intervención intensiva sobre la actividad física Cohorte PREDIMED-Plus” (JS-S); the Recercaixa (grant number 2013ACUP00194) (JS-S). Moreover, JS-S gratefully acknowledges the financial support by ICREA under the ICREA Academia program; the SEMERGEN grant; Department of Health of the Government of Navarra (61/2015), the Fundació La Marató de TV (Ref. 201630.10); the AstraZeneca Young Investigators Award in Category of Obesity and T2D 2017 (DR); grants from the Consejería de Salud de la Junta de Andalucía (PI0458/2013; PS0358/2016; PI0137/2018), the PROMETEO/2017/017 grant from the Generalitat Valenciana, the SEMERGEN grant; grant of support to research groups 35/2011 (Balearic Islands Government; FEDER funds) (JT).

## Acknowledgments

The authors wish to thank the PREDIMED-Plus participants and staff for their engagement, as well as to the primary care centers involved in the study. RS-C acknowledges financial support from the Juan de la Cierva Program Training Grants of the Spanish State Research Agency of the Spanish Ministerio de Ciencia e Innovación y Ministerio de Universidades (FJC2018-038168- I).

## Conflict of interest

JS-S reports grants from CIBEROBN, ISCIII (Spain), during the conduct of the study; non-financial support from Nut and Dried Fruit Foundation, personal fees from Instituto Danone Spain, other from Danone S.A., other from Font Vella Lanjaron, personal fees and grants from Eroski Distributors, grants from Nut and Dried Fruit Foundation, grants from Eroski Distributors, personal fees from Nut and Dried Fruit Foundation, outside the submitted work. ER reports grants, personal fees, non-financial support and other from California Walnut Commission, grants, personal fees, non-financial support and other from Alexion, personal fees, non-financial support and other from Ferrer International, personal fees from Amarin, personal fees, non-financial support and other from Danone, outside the submitted work. JL-M reports personal fees and non-financial support from AMGEN, personal fees and non-financial support from SANOFI, personal fees from MSD, personal fees from Laboratorios Dr. Esteve, personal fees from NOVO-NORDISK outside the submitted work.

## Publisher’s note

All claims expressed in this article are solely those of the authors and do not necessarily represent those of their affiliated organizations, or those of the publisher, the editors and the reviewers. Any product that may be evaluated in this article, or claim that may be made by its manufacturer, is not guaranteed or endorsed by the publisher.
