# Supplementary material for: Morbid liver manifestations are intrinsically bound to metabolic syndrome and nutrient intake based on a machine-learning cluster analysis
Source: Front Endocrinol (Lausanne). 2022 Sep 6;13:936956. doi: 10.3389/fendo.2022.936956 (PMC9487178; doi:10.3389/fendo.2022.936956)
Supplement: Supplementary file 1 [file DataSheet_1.pdf]

Supplementary Table 1: Criteria for the categorization of the variables used in the analysis.

Variable	Categorization	Source
**Waist-Hip Ratio**	< 0.85 (women) < 0.90 (men)	(55)
	≥ 0.85 (women) ≥ 0.90 (men)	
**Hypertension**	Diastolic Pressure < 85 mm Hg - No medicated	(5)
	Diastolic Pressure ≥ 85 mm Hg < 90 mm Hg- No medicated	
	Diastolic Pressure ≥ 90 mm Hg - No medicated	
	Diastolic Pressure < 85 mm Hg - Medicated	
	Diastolic Pressure ≥ 85 mm Hg < 90 mm Hg- Medicated	
	Diastolic Pressure ≥ 90 mm Hg - Medicated	
**Glucose**	Fasting blood glucose levels < 126 mg/dl - No medicated	(5)
	Fasting blood glucose levels ≥ 126 mg/dl - No medicated	
	Fasting blood glucose levels < 126 mg/dl - Medicated	
	Fasting blood glucose levels ≥ 126 mg/dl - Medicated	
**HDL**	Fasting blood HDL levels< 50 mg/dl (women) < 40 mg/dl (men)	(5)
	Fasting blood HDL levels≥ 50 mg/dl (women) ≥ 40 mg/dl (men)	
**LDL**	Fasting blood LDL levels < 100 mg/dl - No medicated	(5)
	Fasting blood LDL levels ≥ 100 mg/dl - No medicated	
	Fasting blood LDL levels < 100 mg/dl - Medicated	
	Fasting blood LDL levels ≥ 100 mg/dl - Medicated	
**Cholesterol**	Fasting blood cholesterol levels < 200 mg/dl - No medicated	(5)
	Fasting blood cholesterol levels < 200 mg/dl - Medicated	
	Fasting blood cholesterol levels ≥ 200 mg/dl - No medicated	
	Fasting blood cholesterol levels ≥ 200 mg/dl - Medicated	
**NLI**	Neutrophils-Lymphocytes Index < 1.35	(56)
	Neutrophils-Lymphocytes Index ≥ 1.35 < 2.25	
	Neutrophils-Lymphocytes Index ≥ 2.25	
**Platelets**	Platelets < 150 (10^3/µL)	(57)
	Platelets ≥ 150 (10^3/µL) < 400 (10^3/µL)	
	Platelets ≥ 400 (10^3/µL)	
**AST**	AST < 8 (UI)	(58)
	AST ≥ 8 (UI) < 33 (UI)	
	AST ≥ 33 (UI)	
**ALT**	ALT < 4 (UI)	(58)
	ALT ≥ 4 (UI) < 36 (UI)	
	ALT ≥ 36 (UI)	
**GGT**	GGT < 5 (UI)	(58)
	GGT ≥ 5 (UI) < 40 (UI)	
	GGT ≥ 40 (UI)	
**Pain treatment**	Presence	-
	Absence	
**Tranquilizers use**	Presence	-
	Absence	
**Insulin use**	Presence	-
	Absence	

**No metformin diabetes treatment**	Presence	-
	Absence	
**CVD medication**	Presence	-
	Absence	
**Aspirin use**	Presence	-
	Absence	

Supplementary Figure 1: Selection of the number of clusters.

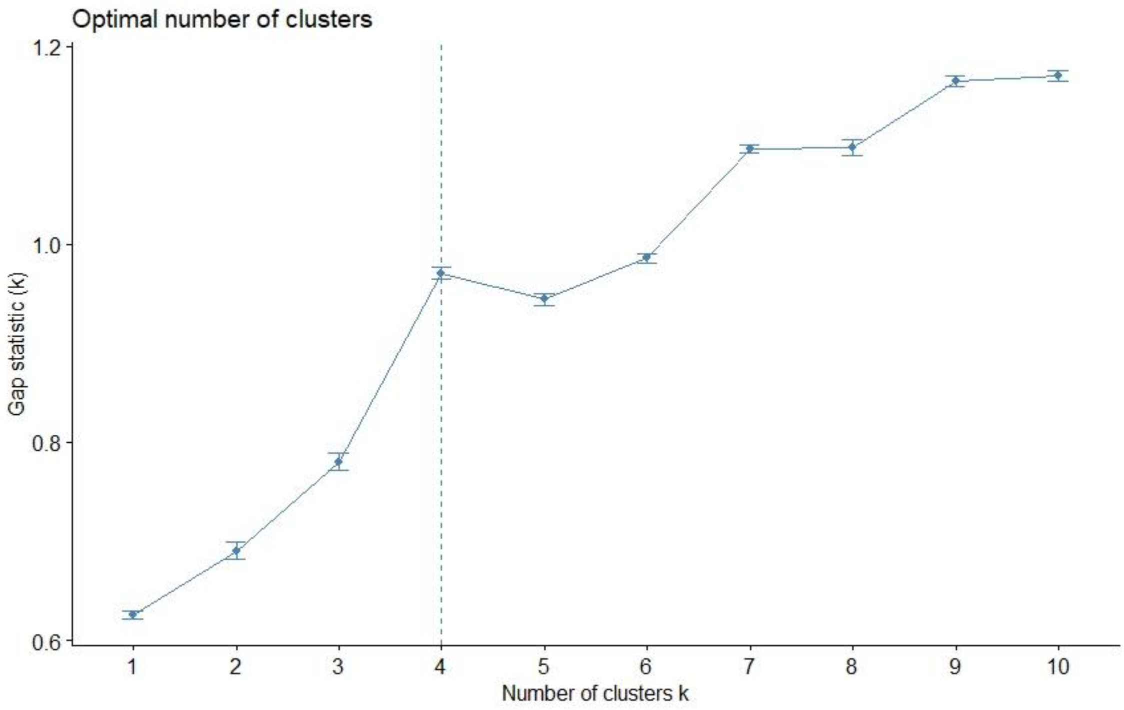

Supplementary table 2. Differences between clusters in pharmacological treatments adjusted by sex, age and recruitment center. between clusters.

	**Cluster 1 (N=616)**	**Cluster 2 (N=1612)**	**Cluster 3 (N=575)**	**Cluster 4 (N=1360)**	***p*-value**
**Hypertension treatment**	521 (84.6%)	1303 (80.8%)	467 (81.2%)	1057 (77.7%)	<0.001
NA	0 (0%)	0 (0%)	0 (0%)	0 (0%)	
**Cholesterol treatment**	444 (72.1%)	1001 (62.1%)	298 (51.8%)	489 (36.0%)	<0.001
NA	0 (0%)	0 (0%)	0 (0%)	0 (0%)	
**Metformin treatmen**	560 (90.9%)	160 (9.9%)	167 (29.0%)	59 (4.3%)	<0.001
**Estatins treatment**	423 (68.7%)	956 (59.3%)	261 (45.4%)	430 (31.6%)	<0.001
**Aspirin treatment**	188 (30.5%)	230 (14.3%)	88 (15.3%)	121 (8.9%)	<0.001
NA	0 (0%)	0 (0%)	0 (0%)	1 (0.1%)	
**Pain treatment**	163 (26.5%)	443 (27.5%)	152 (26.4%)	413 (30.4%)	<0.001
**CVD treatment**	62 (10.1%)	156 (9.7%)	38 (6.6%)	73 (5.4%)	<0.001
NA	0 (0%)	1 (0.1%)	1 (0.2%)	1 (0.1%)	
**No metformin diabetes treatment**	611 (99.2%)	17 (1.1%)	133 (23.1%)	21 (1.5%)	<0.001
**Tranqulizers treatment**	137 (22.2%)	414 (25.7%)	125 (21.7%)	369 (27.1%)	<0.001
NA	0 (0%)	1 (0.1%)	0 (0%)	1 (0.1%)	
**Insulin**	105 (17.0%)	18 (1.1%)	16 (2.8%)	5 (0.4%)	<0.001

p-value refers to ANOVA test comparisons
